# Crystal structure of (OC)_5_W(μ-dppe)W(CO)_5_

**DOI:** 10.1107/S2056989016013670

**Published:** 2016-09-05

**Authors:** Hannah F. Drake, Kraig A. Wheeler, Brian J. Bellott

**Affiliations:** a Department of Chemistry, Western Illinois University, Macomb, Illinois 61455, USA; b Department of Chemistry, Eastern Illinois University, Charleston, Illinois, 61920, USA

**Keywords:** crystal structure, group 6 carbon­yl, bridging dppe, bimetallic

## Abstract

The centrosymmetric title complex, [μ-ethane-1,2-diylbis(di­phenyl­phosphane)-κ^2^ *P*:*P*′]bis­[penta­carbonyl­tungsten(0)], [W_2_(C_26_H_24_P_2_)(CO)_10_], consists of two W(CO)_5_ moieties bridged by a bis­(di­phenyl­phosphan­yl)ethane (dppe) ligand. The W^0^ atom has a slightly distorted octa­hedral coordination environment consisting of 5 carbonyl ligands and one P atom from the bridging dppe ligand with the nearest W^0^ atom 5.625 (5) Å away. The complex resides on a center of symmetry.

## Abstract 2

In the title complex two W(CO)_5_ moieties are bridged by a bis­(di­phenyl­phosphan­yl)ethane (dppe) ligand. Both tungsten atoms have a slightly distorted octa­hedral coordination.

## Chemical context   

In 1976, Pickett and Pletcher studied the mechanism of reduction of a group 6 carbonyl complex in the presence of carbon dioxide (Pickett & Pletcher, 1976 ▸). Recently Grice & Saucedo (2016 ▸) have shown that group 6 metal–carbonyl complexes without ‘non-innocent’ ligands can electrocatalytically reduce CO_2_. Dickson *et al.* (1989 ▸) varied the ligand Ph_2_P(CH_2_)_*n*_PPh_2_ (*n* = 2, 4, and 5), finding that the predominate product in the reactions of *n* = 2 and 5 is the bridged complex (OC)_5_W[μ-Ph_2_P(CH_2_)_*n*_]PPh_2_)W(CO)_5_, whereas when *n* = 4 it was reported the chelated product is favored (W(CO)_4_[μ-Ph_2_P(CH_2_)_4_PPh_2_]. Tan *et al.* (1994 ▸) reported the separation of several diphosphine-bridged group 6 deca­carbonyl complexes by HPLC, but no further characterization was reported. Keiter *et al.* (1981 ▸) and Gan *et al.* (1993 ▸) have reported group 6 heterobimetallic complexes using dppe as the bridging ligand. The title complex has been reported by Keiter & Shah (1972 ▸), Ozer *et al.* (1993 ▸), and El-Khateeb *et al.* (2002 ▸), but the structure has yet to be published. We report here its single crystal X-ray structure determination.
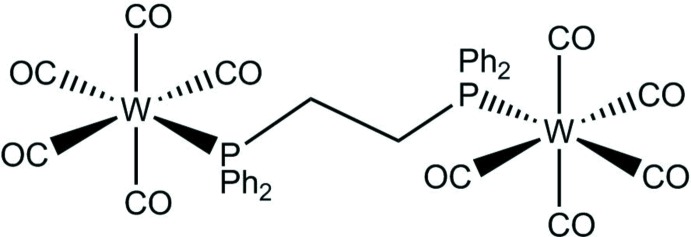


## Structural commentary   

**Figure 1:**
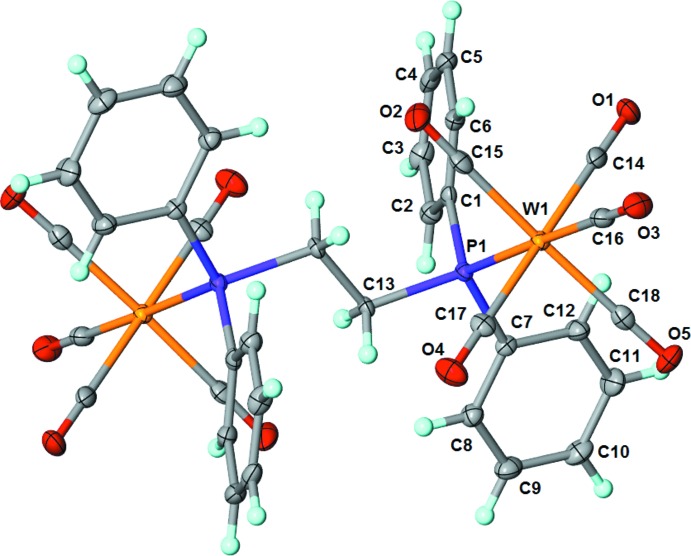
The mol­ecular structure of (OC)_5_W(μ-Ph_2_PCH_2_CH_2_PPh_2_)W(CO)_5_ with displacement ellipsoids drawn at 50% probability level for non-H atoms and H atoms shown as spheres of arbitrary size. Non-labelled atoms are generated by the symmetry operation −*x*, −*y* + 1, −*z* + 1.

The mol­ecular structure of (OC)_5_W(μ-dppe)W(CO)_5_ (Fig. 1 ▸) consists of two six-coordinate tungsten(0) atoms, each in a slightly distorted octa­hedral environment. The coordination environment of tungsten has five carbonyl ligands and one phospho­rus atom from the dppe ligand. The axial carbonyl ligands have a bond length of 2.015 (3) Å and the average bond length for the equatorial carbonyl ligands is 2.048 (8) Å. The W1—P1 bond length is 2.5200 (8) Å and the P1—W1—C(axial) bond angle is 178.79 (9)°. The average P1—W1—C(equatorial) bond angle is 90.10 (18)°. Examination of the dppe backbone shows the P1—C13 bond length at 1.843 (3) Å and the C13—C13 bond length at 1.531 (6) Å. The mol­ecule sits on a center of symmetry.

## Supra­molecular features   

The two tungsten atoms in each of the mol­ecules (OC)_5_W(μ-dppe)W(CO)_5_ are bridged by a diphosphine approximately along the *c* axis and the mol­ecules themselves are stacked along the *a* axis. No significant van der Waals-type inter­actions such as C—H⋯π or π–π contacts between adjacent mol­ecules are observed.

## Database survey   

A search of the database for homonuclear deca­carbonyl group 6 complexes bridged by symmetric phosphines yielded four complexes. There are two tungsten complexes (OC)_5_W[μ-Ph_2_P(CH_2_)_5_PPh_2_]W(CO)_5_ (Ueng & Shih, 1995 ▸), (OC)_5_W(μ-Ph_2_PCH_2_PPh_2_)W(CO)_5_ (Benson *et al.*, 1998 ▸), one molybdenum complex (OC)_5_Mo[μ-Ph_2_P(CH_2_)_2_PPh_2_]Mo(CO)_5_ (Alyea *et al.*, 1990 ▸), and one chromium complex (OC)_5_Cr[μ-Ph_2_P(CH_2_)_5_PPh_2_]Cr(CO)_5_ (Ueng & Shih, 1995 ▸).

## Synthesis and crystallization   

All synthesis and crystallization procedures were carried out using standard Schlenk techniques. Di­chloro­methane was added to a mixture of W(CO)_5_(NH_2_C_6_H_5_) (0.10 g, 2.9 mmol) and dppe (0.12 g, 3.0 mmol) to produce a golden yellow solution. After two h, methanol was added to precipitate a yellow solid. The precipitate was collected and washed with methanol (3 x 20 mL). The resulting yellow solid was recrystallized from a 1:5 mixture of di­chloro­methane:methanol at 253 K.

## Refinement   

Crystal data, data collection, and structure refinement details are summarized in Table 1 ▸. The phenyl H-atom positions and the methyl­ene H atoms on the ligand backbone have been positioned according to idealized C—H distances.

## Supplementary Material

Crystal structure: contains datablock(s) I. DOI: 10.1107/S2056989016013670/vn2114sup1.cif

Structure factors: contains datablock(s) I. DOI: 10.1107/S2056989016013670/vn2114Isup2.hkl

CCDC reference: 1500991

Additional supporting information: crystallographic information; 3D view; checkCIF report

## Figures and Tables

**Table 1:** Experimental details

Crystal data	
Chemical formula	[W_2_(C_26_H_24_P_2_)(CO)_10_]
*M*_r_	1046.17
Crystal system, space group	Monoclinic, *P*2_1_/*n*
Temperature (K)	100
*a*, *b*, *c* (Å)	9.8193 (4), 16.0492 (7), 11.3312 (5)
β (°)	96.511 (2)
*V* (Å^3^)	1774.19 (13)
*Z*	2
Radiation type	Cu *K*α
μ (mm^−1^)	13.15
Crystal size (mm)	0.15 × 0.14 × 0.06

Data collection	
Diffractometer	Bruker APEXII CCD
Absorption correction	Multi-scan (*SADABS*; Bruker, 2011 ▸)
*T*_min_, *T*_max_	0.254, 0.756
No. of measured, independent and observed [*I* > 2σ(*I*)] reflections	26419, 3256, 2954
*R*_int_	0.056
(sin θ/λ)_max_ (Å^−1^)	0.602

Refinement	
*R*[*F* ^2^ > 2σ(*F* ^2^)], *wR*(*F* ^2^), *S*	0.021, 0.048, 1.06
No. of reflections	3256
No. of parameters	226
H-atom treatment	H-atom parameters constrained
Δρ_max_, Δρ_min_ (e Å^−3^)	0.54, −0.58

Computer programs: *APEX2* and *SAINT* (Bruker, 2011 ▸), *SHELXT* (Sheldrick, 2015*a* ▸), *SHELXL2014* (Sheldrick, 2015*b* ▸) and *X-SEED* (Barbour, 2001 ▸).

## supplementary crystallographic information

### Crystal data

**Table:**

[W_2_(C_26_H_24_P_2_)(CO)_10_]	*F*(000) = 996
*M**_r_* = 1046.17	*D*_x_ = 1.958 Mg m^−^^3^
Monoclinic, *P*2_1_/*n*	Cu *K*α radiation, λ = 1.54178 Å
*a* = 9.8193 (4) Å	Cell parameters from 8094 reflections
*b* = 16.0492 (7) Å	θ = 4.6–66.6°
*c* = 11.3312 (5) Å	µ = 13.15 mm^−^^1^
β = 96.511 (2)°	*T* = 100 K
*V* = 1774.19 (13) Å^3^	Transparent rhomboid, colorless
*Z* = 2	0.15 × 0.14 × 0.06 mm

### Data collection

**Table:**

Bruker APEXII CCD diffractometer	2954 reflections with *I* > 2σ(*I*)
Detector resolution: 8.33 pixels mm^-1^	*R*_int_ = 0.056
phi and ω scans	θ_max_ = 68.2°, θ_min_ = 4.8°
Absorption correction: multi-scan (SADABS; Bruker, 2011)	*h* = −11→11
*T*_min_ = 0.254, *T*_max_ = 0.756	*k* = −19→19
26419 measured reflections	*l* = −13→13
3256 independent reflections	

### Refinement

**Table:**

Refinement on *F*^2^	0 restraints
Least-squares matrix: full	Hydrogen site location: inferred from neighbouring sites
*R*[*F*^2^ > 2σ(*F*^2^)] = 0.021	H-atom parameters constrained
*wR*(*F*^2^) = 0.048	*w* = 1/[σ^2^(*F*_o_^2^) + (0.0218*P*)^2^ + 0.5664*P*] where *P* = (*F*_o_^2^ + 2*F*_c_^2^)/3
*S* = 1.06	(Δ/σ)_max_ = 0.001
3256 reflections	Δρ_max_ = 0.54 e Å^−^^3^
226 parameters	Δρ_min_ = −0.58 e Å^−^^3^

### Special details

**Table:**

Geometry. All esds (except the esd in the dihedral angle between two l.s. planes) are estimated using the full covariance matrix. The cell esds are taken into account individually in the estimation of esds in distances, angles and torsion angles; correlations between esds in cell parameters are only used when they are defined by crystal symmetry. An approximate (isotropic) treatment of cell esds is used for estimating esds involving l.s. planes.
Refinement. All nonhydrogen atoms were located in a single difference Fourier electron density maps and refined using anisotropic diplacement parameters. All C-H hydrogen atoms were placed in calculated positions with Uiso = 1.2xUeqiv of the connected C atoms

### Fractional atomic coordinates and isotropic or equivalent isotropic displacement parameters (Å^2^)

**Table:**

	*x*	*y*	*z*	*U*_iso_*/*U*_eq_	
W1	0.17092 (2)	0.47944 (2)	0.81902 (2)	0.01462 (6)	
P1	0.15114 (8)	0.56563 (5)	0.63197 (7)	0.01359 (16)	
O1	0.1345 (2)	0.64362 (16)	0.9701 (2)	0.0277 (6)	
O2	−0.1519 (2)	0.44991 (18)	0.7958 (2)	0.0324 (6)	
O3	0.2025 (2)	0.36975 (16)	1.0520 (2)	0.0286 (6)	
O4	0.2103 (3)	0.31759 (16)	0.6641 (2)	0.0317 (6)	
O5	0.4947 (2)	0.50864 (15)	0.8560 (2)	0.0274 (6)	
C1	0.0518 (3)	0.66234 (19)	0.6298 (3)	0.0162 (6)	
C2	0.0733 (3)	0.7262 (2)	0.5503 (3)	0.0206 (7)	
H2	0.1383	0.7188	0.4950	0.025*	
C3	0.0010 (3)	0.8002 (2)	0.5510 (3)	0.0251 (8)	
H3	0.0155	0.8429	0.4958	0.030*	
C4	−0.0924 (3)	0.8119 (2)	0.6323 (3)	0.0253 (8)	
H4	−0.1402	0.8633	0.6342	0.030*	
C5	−0.1164 (3)	0.7490 (2)	0.7106 (3)	0.0238 (7)	
H5	−0.1813	0.7571	0.7657	0.029*	
C6	−0.0453 (3)	0.6737 (2)	0.7089 (3)	0.0196 (7)	
H6	−0.0633	0.6301	0.7618	0.023*	
C7	0.3146 (3)	0.60406 (19)	0.5907 (3)	0.0149 (6)	
C8	0.3683 (3)	0.5823 (2)	0.4868 (3)	0.0202 (7)	
H8	0.3191	0.5455	0.4319	0.024*	
C9	0.4940 (3)	0.6143 (2)	0.4630 (3)	0.0264 (8)	
H9	0.5300	0.5991	0.3917	0.032*	
C10	0.5671 (3)	0.6679 (2)	0.5420 (3)	0.0260 (8)	
H10	0.6532	0.6892	0.5256	0.031*	
C11	0.5130 (4)	0.6903 (2)	0.6460 (3)	0.0269 (8)	
H11	0.5614	0.7280	0.7001	0.032*	
C12	0.3894 (3)	0.6578 (2)	0.6706 (3)	0.0218 (7)	
H12	0.3546	0.6722	0.7428	0.026*	
C13	0.0738 (3)	0.51389 (19)	0.4953 (3)	0.0146 (6)	
H13A	0.0747	0.5528	0.4275	0.018*	
H13B	0.1297	0.4647	0.4793	0.018*	
C14	0.1472 (3)	0.5850 (2)	0.9158 (3)	0.0205 (7)	
C15	−0.0363 (4)	0.4607 (2)	0.8030 (3)	0.0213 (7)	
C16	0.1906 (3)	0.4096 (2)	0.9678 (3)	0.0207 (7)	
C17	0.1959 (3)	0.3754 (2)	0.7191 (3)	0.0202 (7)	
C18	0.3793 (4)	0.4984 (2)	0.8413 (3)	0.0205 (7)	

### Atomic displacement parameters (Å^2^)

**Table:**

	*U*^11^	*U*^22^	*U*^33^	*U*^12^	*U*^13^	*U*^23^
W1	0.01547 (9)	0.01626 (9)	0.01235 (9)	−0.00103 (5)	0.00255 (5)	0.00059 (6)
P1	0.0139 (4)	0.0142 (4)	0.0128 (4)	−0.0021 (3)	0.0019 (3)	−0.0009 (3)
O1	0.0315 (14)	0.0288 (14)	0.0221 (13)	0.0051 (11)	−0.0009 (10)	−0.0088 (12)
O2	0.0196 (14)	0.0540 (18)	0.0243 (13)	−0.0101 (12)	0.0050 (10)	0.0027 (13)
O3	0.0304 (14)	0.0356 (15)	0.0209 (13)	0.0049 (11)	0.0071 (10)	0.0106 (12)
O4	0.0430 (16)	0.0231 (14)	0.0311 (14)	−0.0061 (11)	0.0128 (12)	−0.0054 (12)
O5	0.0162 (13)	0.0269 (13)	0.0380 (15)	−0.0008 (10)	−0.0017 (10)	0.0032 (12)
C1	0.0159 (15)	0.0160 (15)	0.0157 (16)	−0.0016 (12)	−0.0030 (12)	−0.0033 (13)
C2	0.0194 (16)	0.0193 (17)	0.0230 (17)	0.0002 (13)	0.0012 (13)	−0.0001 (15)
C3	0.0227 (17)	0.0193 (17)	0.0319 (19)	−0.0013 (14)	−0.0028 (14)	0.0053 (16)
C4	0.0167 (16)	0.0219 (17)	0.035 (2)	0.0033 (14)	−0.0070 (14)	−0.0069 (16)
C5	0.0157 (16)	0.0307 (19)	0.0244 (18)	0.0028 (14)	0.0003 (13)	−0.0076 (16)
C6	0.0157 (15)	0.0255 (18)	0.0170 (16)	0.0007 (13)	0.0001 (13)	−0.0011 (14)
C7	0.0159 (15)	0.0133 (15)	0.0158 (15)	−0.0010 (12)	0.0029 (12)	0.0043 (13)
C8	0.0186 (16)	0.0198 (17)	0.0224 (17)	−0.0024 (13)	0.0027 (13)	0.0000 (14)
C9	0.0210 (17)	0.033 (2)	0.0259 (18)	0.0012 (15)	0.0080 (14)	0.0037 (17)
C10	0.0179 (17)	0.0234 (18)	0.037 (2)	−0.0029 (14)	0.0057 (15)	0.0068 (17)
C11	0.0228 (18)	0.0254 (18)	0.032 (2)	−0.0090 (14)	0.0025 (15)	−0.0033 (17)
C12	0.0184 (16)	0.0236 (18)	0.0243 (18)	−0.0023 (14)	0.0063 (13)	−0.0030 (15)
C13	0.0177 (16)	0.0152 (15)	0.0109 (15)	−0.0028 (12)	0.0007 (12)	−0.0027 (13)
C14	0.0175 (16)	0.0303 (19)	0.0136 (16)	0.0017 (13)	0.0016 (13)	0.0012 (15)
C15	0.029 (2)	0.0228 (18)	0.0132 (16)	−0.0016 (14)	0.0055 (13)	0.0010 (14)
C16	0.0192 (16)	0.0230 (17)	0.0209 (18)	0.0021 (13)	0.0062 (13)	−0.0015 (16)
C17	0.0241 (17)	0.0188 (17)	0.0186 (17)	−0.0033 (13)	0.0067 (13)	0.0024 (15)
C18	0.029 (2)	0.0147 (16)	0.0180 (17)	−0.0001 (14)	0.0025 (14)	0.0002 (14)

### Geometric parameters (Å, º)

**Table:**

W1—C16	2.015 (3)	C4—C5	1.381 (5)
W1—C15	2.044 (4)	C4—H4	0.9500
W1—C14	2.045 (4)	C5—C6	1.398 (5)
W1—C17	2.048 (3)	C5—H5	0.9500
W1—C18	2.056 (4)	C6—H6	0.9500
W1—P1	2.5200 (8)	C7—C8	1.388 (4)
P1—C7	1.829 (3)	C7—C12	1.397 (5)
P1—C1	1.832 (3)	C8—C9	1.391 (5)
P1—C13	1.843 (3)	C8—H8	0.9500
O1—C14	1.140 (4)	C9—C10	1.382 (5)
O2—C15	1.143 (4)	C9—H9	0.9500
O3—C16	1.144 (4)	C10—C11	1.394 (5)
O4—C17	1.135 (4)	C10—H10	0.9500
O5—C18	1.139 (4)	C11—C12	1.378 (5)
C1—C6	1.393 (4)	C11—H11	0.9500
C1—C2	1.397 (5)	C12—H12	0.9500
C2—C3	1.384 (5)	C13—C13^i^	1.531 (6)
C2—H2	0.9500	C13—H13A	0.9900
C3—C4	1.385 (5)	C13—H13B	0.9900
C3—H3	0.9500		
			
C16—W1—C15	89.55 (13)	C4—C5—H5	119.9
C16—W1—C14	91.03 (13)	C6—C5—H5	119.9
C15—W1—C14	89.83 (13)	C1—C6—C5	120.1 (3)
C16—W1—C17	90.16 (13)	C1—C6—H6	120.0
C15—W1—C17	90.64 (13)	C5—C6—H6	120.0
C14—W1—C17	178.72 (13)	C8—C7—C12	118.8 (3)
C16—W1—C18	88.85 (13)	C8—C7—P1	124.1 (2)
C15—W1—C18	178.03 (12)	C12—C7—P1	117.0 (2)
C14—W1—C18	89.05 (13)	C7—C8—C9	120.2 (3)
C17—W1—C18	90.52 (13)	C7—C8—H8	119.9
C16—W1—P1	178.79 (9)	C9—C8—H8	119.9
C15—W1—P1	91.45 (9)	C10—C9—C8	120.7 (3)
C14—W1—P1	89.64 (9)	C10—C9—H9	119.6
C17—W1—P1	89.16 (9)	C8—C9—H9	119.6
C18—W1—P1	90.16 (9)	C9—C10—C11	119.2 (3)
C7—P1—C1	101.08 (14)	C9—C10—H10	120.4
C7—P1—C13	103.13 (14)	C11—C10—H10	120.4
C1—P1—C13	101.69 (14)	C12—C11—C10	120.2 (3)
C7—P1—W1	114.44 (10)	C12—C11—H11	119.9
C1—P1—W1	117.80 (11)	C10—C11—H11	119.9
C13—P1—W1	116.38 (10)	C11—C12—C7	120.8 (3)
C6—C1—C2	118.9 (3)	C11—C12—H12	119.6
C6—C1—P1	120.3 (2)	C7—C12—H12	119.6
C2—C1—P1	120.7 (2)	C13^i^—C13—P1	112.1 (3)
C3—C2—C1	120.7 (3)	C13^i^—C13—H13A	109.2
C3—C2—H2	119.6	P1—C13—H13A	109.2
C1—C2—H2	119.6	C13^i^—C13—H13B	109.2
C2—C3—C4	120.0 (3)	P1—C13—H13B	109.2
C2—C3—H3	120.0	H13A—C13—H13B	107.9
C4—C3—H3	120.0	O1—C14—W1	179.7 (3)
C5—C4—C3	120.1 (3)	O2—C15—W1	179.0 (3)
C5—C4—H4	119.9	O3—C16—W1	179.6 (3)
C3—C4—H4	119.9	O4—C17—W1	179.6 (3)
C4—C5—C6	120.2 (3)	O5—C18—W1	178.7 (3)
			
C7—P1—C1—C6	−146.7 (3)	W1—P1—C7—C8	117.1 (3)
C13—P1—C1—C6	107.2 (3)	C1—P1—C7—C12	65.5 (3)
W1—P1—C1—C6	−21.3 (3)	C13—P1—C7—C12	170.5 (3)
C7—P1—C1—C2	32.5 (3)	W1—P1—C7—C12	−62.2 (3)
C13—P1—C1—C2	−73.6 (3)	C12—C7—C8—C9	−0.5 (5)
W1—P1—C1—C2	157.9 (2)	P1—C7—C8—C9	−179.7 (3)
C6—C1—C2—C3	1.0 (5)	C7—C8—C9—C10	0.0 (5)
P1—C1—C2—C3	−178.1 (3)	C8—C9—C10—C11	−0.5 (5)
C1—C2—C3—C4	0.9 (5)	C9—C10—C11—C12	1.4 (5)
C2—C3—C4—C5	−1.7 (5)	C10—C11—C12—C7	−1.8 (5)
C3—C4—C5—C6	0.7 (5)	C8—C7—C12—C11	1.4 (5)
C2—C1—C6—C5	−2.1 (5)	P1—C7—C12—C11	−179.4 (3)
P1—C1—C6—C5	177.1 (2)	C7—P1—C13—C13^i^	−172.8 (3)
C4—C5—C6—C1	1.2 (5)	C1—P1—C13—C13^i^	−68.3 (3)
C1—P1—C7—C8	−115.3 (3)	W1—P1—C13—C13^i^	61.0 (3)
C13—P1—C7—C8	−10.3 (3)		

Symmetry code: (i) −*x*, −*y*+1, −*z*+1.
